# Cancer Care Partners’ Transitions in Self-identity from Pre-Cancer into Survivorship: A Mixed Methods Analysis

**DOI:** 10.1093/geroni/igaf122.798

**Published:** 2025-12-31

**Authors:** Ann Kuglin Jones, Lee Ellington, Linda Edelman, Lanell Bellury, Darryl Somayaji, Kristin Cloyes

**Affiliations:** Wings of Hope Cancer Support Center, Council Bluffs, Iowa, United States; University of Utah, Salt Lake City, Utah, United States; University of Utah, Salt Lake City, Utah, United States; Mercer University, Macon, Georgia, United States; University of Buffalo, Buffalo, New York, United States; Oregon Health & Science University, Portland, Oregon, United States

## Abstract

Cancer survivors’ transitions from diagnosis into post-treatment life are well-described. Care partners’ transition experiences, however, remain underrepresented. Our mixed methods study combined NLP and qualitative analysis to examine shifts in care partners’ self-identity from pre-cancer into survivorship. We enrolled n = 18 spouses/partners (n = 9 women, n = 9 men) of post-treatment survivors. Participants completed two free listing exercises and two interviews describing “who am I” before and after cancer. Selected LIWC linguistic markers measures identity-related and affective content, and Wilcoxon signed-rank and Mann-Whitney U tests compared differences pre/post cancer and by gender. Abductive interview coding, integrating transition theory concepts and inductive analysis, identified themes. When describing their before-cancer self-identity, participants used more positive (Mdn = 96.94 vs. 54.60, p = .025) and self-focused language (Mdn = 0.50 vs. 3.50, p = .008). Post-cancer descriptions were more relational in focus with more “we” language (Mdn = 0.00 vs. 1.00, p = .009) with more negative affect, including anxiety (p = .019), and sadness (p = .043). Described changes in self-identity evoked uncertainty, role ambiguity, evolving relationship dynamics, and dissatisfaction with available identity labels. Participants rejected “caregiver’ as too unidirectional and “survivor” as inapt, while also describing their experiences a life- and identity-changing transition. Care partners lack a shared, recognized vocabulary to articulate their survivorship experiences, challenging their ability to find meaning in and connect with current survivorship discourse. Health care providers can support and validate care partners’ identities through person-centered communication that uses their language, acknowledges their unique experiences, and assesses their unique support needs.

